# Deposits from evaporating emulsion drops

**DOI:** 10.1038/s41598-020-71964-1

**Published:** 2020-09-10

**Authors:** M. R. Bittermann, A. Deblais, S. Lépinay, D. Bonn, N. Shahidzadeh

**Affiliations:** Van der Waals-Zeeman Institute, IoP, Science Park 904, Amsterdam, Netherlands

**Keywords:** Soft materials, Fluids, Wetting

## Abstract

The processes in which droplets evaporate from solid surfaces, leaving behind distinct deposition patterns, have been studied extensively for variety of solutions. In this work, by combining different microscopy techniques (confocal fluorescence, video and Raman) we investigate pattern formation and evaporation-induced phase change in drying oil-in-water emulsion drops. This combination of techniques allows us to perform drop shape analysis while visualizing the internal emulsion structure simultaneously. We observe that drying of the continuous water phase of emulsion drops on hydrophilic surfaces favors the formation of ring-like zones depleted of oil droplets at the contact line, which originate from geometrical confinement of oil droplets by the meniscus. From such a depletion zone, a “coffee ring” composed of surfactant molecules forms as the water evaporates. On all surfaces drying induces emulsion destabilization by coalescence of oil droplets, commencing at the drop periphery. For hydrophobic surfaces, the coalescence of the oil droplets leads to a uniform oil film spreading out from the initial contact line. The evaporation dynamics of these composite drops indicate that the water in the continuous phase of the emulsion drops evaporates predominantly by diffusion through the vapor, showing no large differences to the evaporation of simple water drops.

## Introduction

One of the most ubiquitous yet intriguing features of drying drops is evaporation-induced pattern formation on the substrate, of which the coffee-ring effect, where solute is deposited at the contact line, is the most prominent example^1–9^. However, the accumulation of deposits at the drop periphery can be undesired in the fields of inkjet printing^10,11^, microdot arrays^12^, biotechnological applications^13^ and other coating and patterning technologies, in which a high degree of deposit uniformity is crucial. To solve this problem, extensive research over the past decades has been dedicated towards finding means to control dried drop morphologies, for instance by capillary forces^14,15^, Marangoni flows^16,17^, or the addition of surfactants^18–20^. Many of these studies focus on deposition of solid colloidal particles from drying solutions^11,18,21,22^, whereas for a large number of applications the drops are in fact much more complex than a simple suspension. One more complicated but relevant system is that of emulsions, where the “particles” are liquid droplets, which is what we study here. Previous works show that emulsion drops can lead to complex evaporation scenarios and undergo drying-induced phase changes; however these studies were limited to rather low volume fractions^23,24^.

Here, by combining confocal fluorescence microscopy and video microscopy we study pattern formation and its associated dynamics of drying concentrated oil-in-water (o/w) emulsion drops. Our emulsions are ternary mixtures of oil, water and surfactant, in which only the aqueous component evaporates. As a simple model system we consider an o/w emulsion with dispersed non-volatile silicon oil droplets in water with an oil volume fraction of $\phi = 0.60$, which is below $\phi _c \sim 0.64$, the volume fraction giving rise to the jamming transition for simple yield stress fluids^25^. We choose $\phi < \phi _c$ so that the drops do not exhibit a yield stress, allowing to have a good control of the initial shape of the drop when deposited on the substrate. The droplets are stabilized by the ionic surfactant sodium dodecyl sulfate (SDS) at 1% mass fraction^26^ and are of $10 \, \upmu \hbox {m}$ diameter in average. Thermodynamics always drives emulsions towards destabilization, i.e. phase separation between the water and oil phase. We observe here that the evaporation of the volatile water phase of the emulsion favors coalescence of the dispersed non-volatile oil phase, leading to a drying-induced phase separation of water and oil^27–29^. By adjusting the wettability of the substrate the emulsion drops are resting on, we find that the final morphology of dried emulsion drops can be controlled by changing the initial contact angle. Complete evaporation of the water phase on hydrophilic substrates (low contact angles) leaves behind ring-like shapes at the drop periphery, which we identify as depletion zones, induced by geometrical confinement of oil droplets at the contact line. This effect can be completely suppressed by drying the emulsion drops on hydrophobic surfaces instead, where the initial contact angle is high and the oil drops are not confined near the contact line. Then, upon destabilization of the oil droplets on the hydrophobic substrate, an oil film is released, which uniformly coats the substrate. Moreover, we find that not only the drop morphology but also the evaporation speed depends on the wetting properties of the substrate. The evaporation rate increases when the contact angle decreases and remarkably the drying rates can be described by simple diffusion laws for the water vapor.

**Figure 1:**
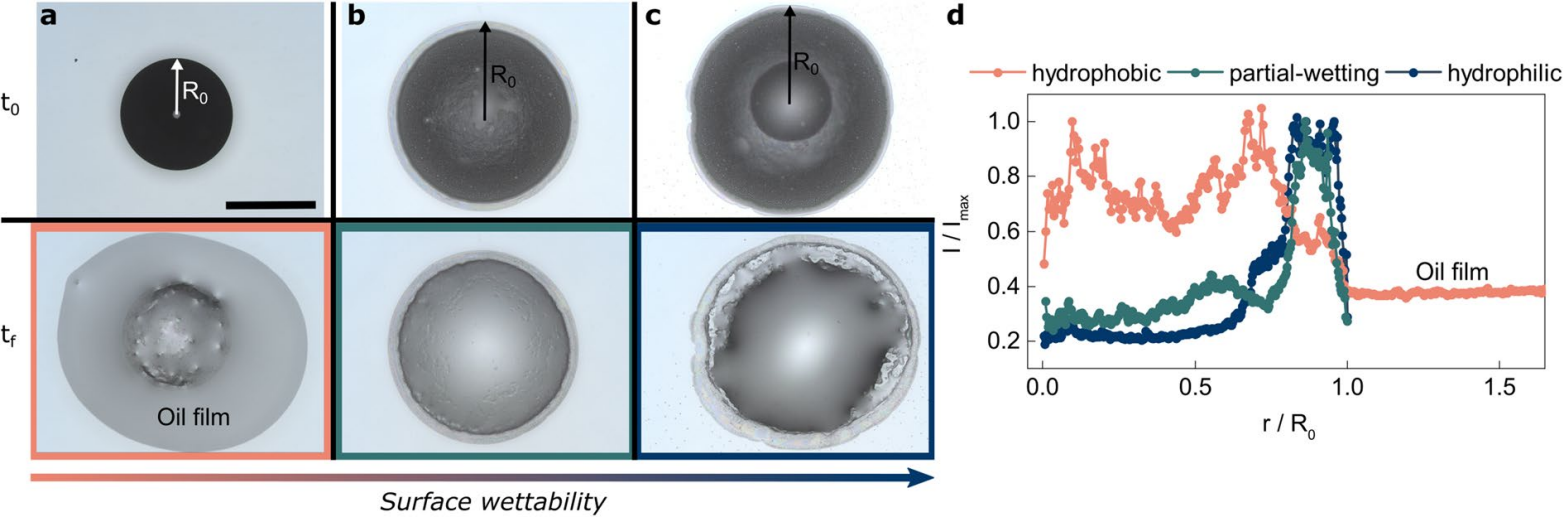
Final deposits after drying of emulsion drops on surfaces with variable surface wettability. Optical microscopy images of emulsion drops after deposition ($t_0$) and complete evaporation ($t_f$) on (**a**) hydrophobic, (**b**) partial-wetting and (**c**) hydrophilic surfaces. The surface wettability increases from hydrophobic to hydrophilic for both water and oil phase. Scale bar is 1.2 mm. (**d**) Radial intensity profiles of dried emulsion drops calculated via Sobel edge-detection, highlighting non-uniformities within the deposition profiles. Ring-like shapes on the drop periphery occur on both wetting surfaces, whilst being absent on the hydrophobic one, where protrusions on the drop surfaces appear in the center. As evident from the microscopy images, the dried drop on the hydrophobic substrate reaches beyond its initial diameter, due to an oil film spreading on the surface. Conversely, on wetting substrates the drop diameters remain unchanged upon drying.

## Results

### Final morphology of drying emulsion drops on different substrates

In a first experiment we study how the final morphology of dried oil-in-water emulsion drops is influenced by the wettability of the surface (towards both water phase and oil, see Materials and Methods section for the initial contact angles) the drops are deposited on. We use a Keyence optical profilometer to follow the drying emulsion drops transition from their wet state right after deposition (time $t_0$) to their final state (time $t_f$). After complete evaporation of the water phase, the drop leaves behind the non-volatile oil and the equally non-volatile surfactant. The differences in drop morphology between initial and final states on substrates of different wettability are shown in Fig. 1a–c (top and bottom panel for $t_0$ and $t_f$, respectively). Initially, the drops appear black because the oil droplets scatter a lot of light. When the water evaporates, the drops become light grey on all surfaces; the scattering from the drops decreases, suggesting that the evaporation induces coalescence of dispersed oil droplets. Looking at the final drop shapes, evaporation from the silanized hydrophobic surface (Fig. 1a) results in a deposition pattern at $t_f$, which reveals that an oil film is released, spreading beyond the initial drop diameter and uniformly covering the glass slide. Evaporation from untreated (partial-wetting) glass slides however, leaves ring-like shapes on the periphery (Fig. 1b), an effect which can be enhanced by increasing the wettability further via plasma-treatment of the glass surface (Fig. 1c).

We evaluate the profiles of the final deposition patterns using a Sobel edge-detection algorithm, which highlights structural non-uniformities (see Materials and Methods section for more detail). For the hydrophobic substrate, Fig. 1d shows heterogeneities in the drop center, and a homogeneous oil film spreading far beyond the initial drop diameter. On the other hand, for the two more hydrophilic substrates, we observe peaks at the dried emulsion drop periphery. These coffee-ring type deposits take up 20 and 30% of the whole drop diameter for the partial-wetting and hydrophilic surfaces, respectively. Given that SDS is practically insoluble in the oil phase (hydrophile lipophile balance number $\sim$ 40), we surmise any structural heterogeneities to be crystalline SDS. To corroborate this claim we carried out Raman microscopy measurements (see Fig. S1 in the Supplementary Information) on both drop periphery and central drop area. We indeed find spectral fingerprints of crystalline SDS to be present within the coffee stains for wetting surfaces, together with a small amount of oil. On the hydrophobic surface, we detect SDS in the center of the main drop, explaining the protrusions visible in Fig. 1a.

This first experiment indicates that three distinct mechanisms occur as a consequence of the evaporating water phase: coalescence of the oil droplets, precipitation of crystals of the surfactant SDS and formation of a ring-like shape at the drop periphery on the more hydrophilic surfaces. The following sections will be dedicated towards elucidating dynamics and mechanisms involved in the drying processes leading up to the individual deposits.

**Figure 2:**
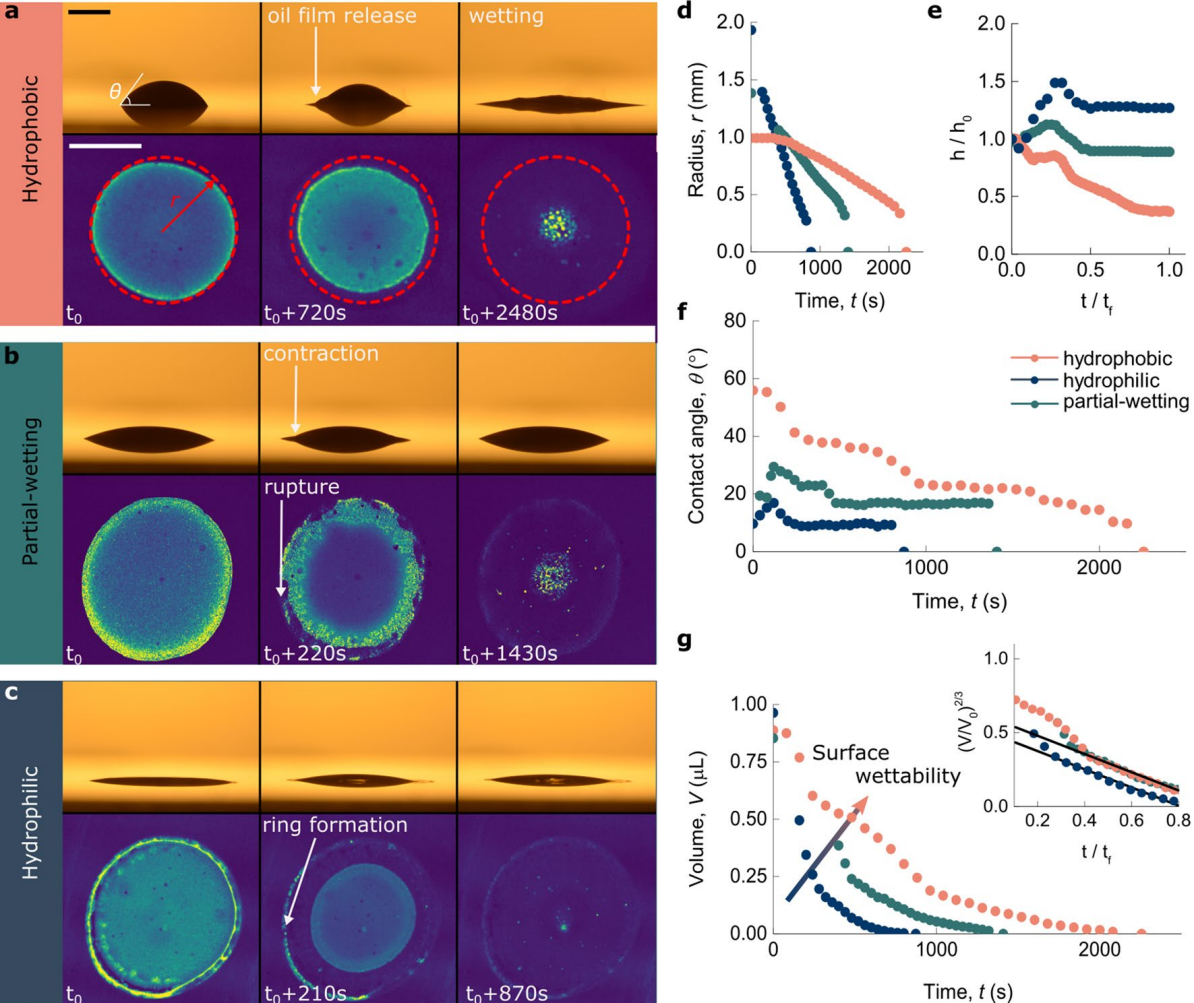
Evaporation dynamics of o/w emulsion drops. (**a**–**c**) Snapshots of drying emulsion drops on (**a**) hydrophobic, (**b**) partial-wetting, and (**c**) hydrophilic surfaces. The upper panels show the evolution of the emulsion drop shape in time (side views) and the lower panels the corresponding fluorescence images (bottom views) of the stained aqueous phase. From these measurements we deduce the temporal evolution of the drop morphology (radius, height, contact angle) and infer the internal structure of the drop in terms of evaporation dynamics and destabilisation mechanisms. Scale bar is $160 \, \upmu \hbox {m}$. (**d**) Radius *r* of the continuous phase of the drying drops as a function of time, measured via confocal microscopy. The radius at any given time is calculated from the perimeter of the evaporating continuous phase (highlighted by the dashed red circle in (**a**)). On the hydrophobic surface, the radius initially remains constant and evaporation commences in the so-called constant contact radius (CCR) regime. For hydrophilic and partial-wetting surfaces there is no pinning and the radii decrease linearly. (**e**) Normalized height evolution, obtained from video microscopy images from the side. Data points were rescaled by the final evaporation time $t_{f}$ to improve readability. Drying on both more hydrophilic substrates causes the global drop height to first increase as the radius of the water phase decreases, followed by constant plateaus. The effect is most pronounced on the hydrophilic glass slide. On the silanized substrate the drop height initially decreases, followed by a constant plateau, before decreasing again. (**f**) Contact angle $\theta$ as a function of time. Emulsion drops on wetting surfaces dry predominantly in constant contact angle (CCA) mode, except for short sections corresponding to the height increase shown in (**e**). Evaporation on hydrophobic surfaces however reveal a more complicated picture. CCA regimes are separated by sudden changes in the contact angle. We refer to this evaporation scenario as mixed mode. (**g**) Droplet volume *V* (calculated as described in the Supplementary Information) as a function of drying time for the three surfaces investigated here. The inset shows a plot of $(V/{V_{0}})^\frac{2}{3}$ versus $t/t_f$. The linear regions starting around $0.4 \, t_f$ hint at diffusion-controlled evaporation processes.

### Dynamics of evaporating emulsion drops

Given the large differences in the final deposits of the dried drops on surfaces of different wettability, we carried out confocal fluorescence microscopy looking at the emulsion stucture in 3d, coupled with video microscopy to have a side view of the evaporating drops. This allows to study the underlying mechanisms and dynamics involved in the evaporation and coalescence processes. The fluorescent dye Rhodamine 6G was dissolved in the continuous water phase and excited with laser light from an inverted confocal microscope. The time evolution of the drying drops is shown in Fig. 2. The bottom panels show confocal microscopy images from below and the top panels show the simultaneously taken video microscopy images from the side ($t_0 \sim 100$ s after deposition).

**Figure 3:**
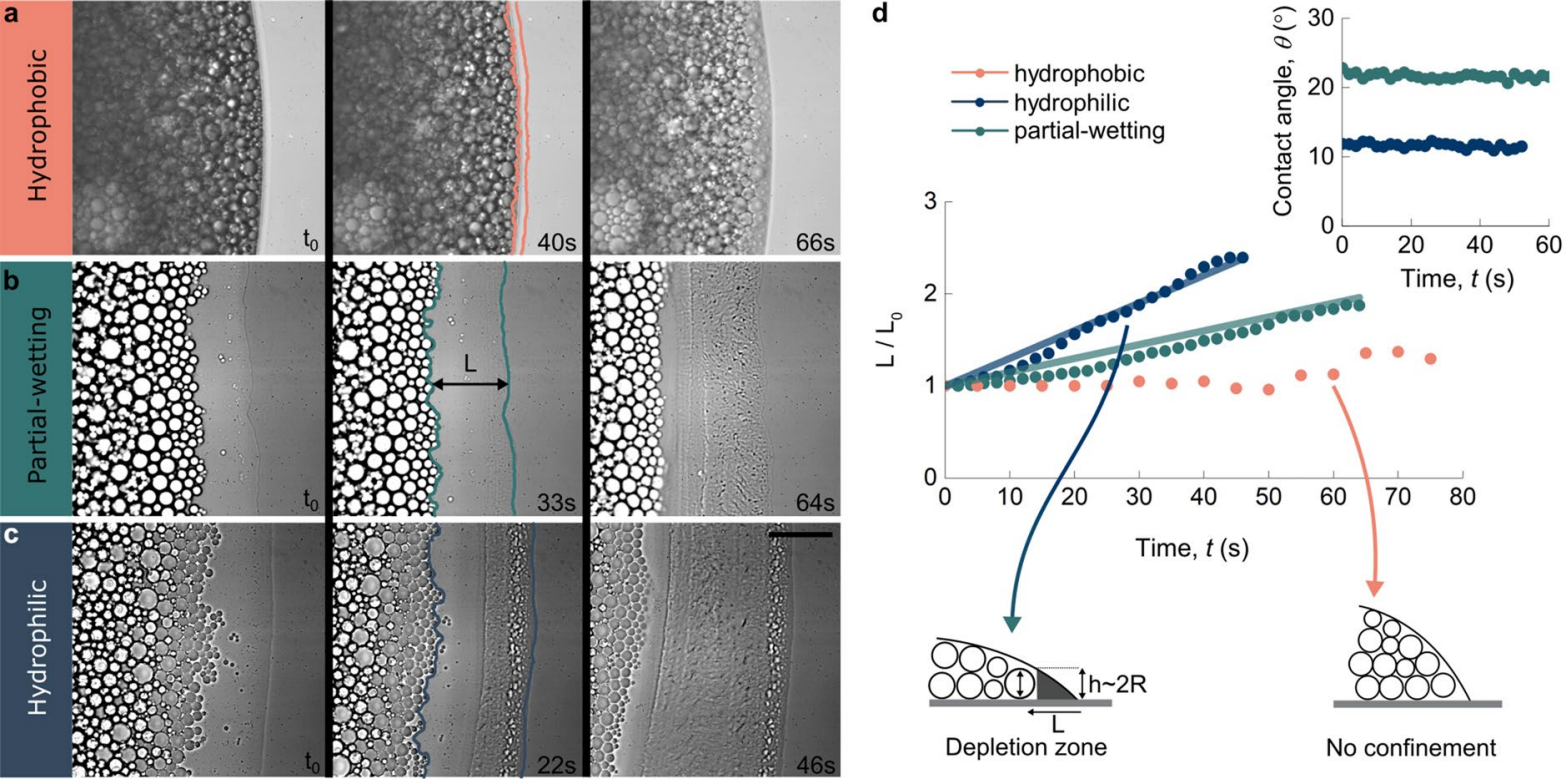
Dynamics of the receding dispersed phase. (**a**–**c**) Microscopy snapshots of areas close to the contact line. Zooming in on the emulsion drop periphery reveals oil droplet displacements prior to phase inversion. This effect prevails on wetting surfaces [(**b**, **c**) for partial-wetting and hydrophilic surfaces, respectively] and is nearly absent on the hydrophobic surface (**a**). As destabilization sets in, the droplets “freeze” and their movement halts. Scale bar is $75 \, \upmu \hbox {m}$ (**d**) Width of the normalized depletion zone *L*, as indicated in (**b**), as a function of drying time for all surfaces. The schematic drawings illustrate the conditions needed for depletion, i.e. the solution film thickness *h* falling below the diameter 2*R* of the individual oil particles. The transparent lines show linear fits $L/L_0 \propto (a/\theta )\, t$ based on a global fitting parameter *a*, which depends on the droplet size. Inset shows constant contact angles for the initial regime for both wetting substrates.

Looking at evaporating emulsion drops on the hydrophobic substrate, we observe contact line pinning, as the radius remains constant while height and the initial contact angle of $\sim 56^{\circ }$ decrease (Fig. 2a bottom panel,d–f). This regime is commonly refereed to as constant contact radius (CCR) mode evaporation^4^. After some time the water-rich phase starts to retract and we observe constant contact angle (CCA) evaporation to be interrupted by abrupt changes in the contact angle during drying, giving rise to a more complex evaporation scenario, which we hereby refer to as mixed evaporation mode. The onset of the mixed mode is accompanied by the formation of the oil layer on the surface (Fig. 2a top panel). On the partial-wetting and hydrophilic surfaces the initial contact angles are reduced to values of $\sim 23^{\circ }$ and $\sim 10^{\circ }$, respectively (Fig. 2f). Here, the radii of the continuous phases shrink immediately after drop deposition, and evaporation occurs predominantly in CCA mode. Additionally, the drops rupture and start to separate in different phases near the drop edge leaving behind the coffee ring-like shape already discussed above (Fig. 1b–d). Following the formation of the ring-like shapes, the emulsion drops contract, as the height at their centers increases along with their contact angles (Fig.2b,c top panel,e,f). Incorporating contact angles and base radii of the continuous phase into a spherical cap model (see Supplementary Information) allows us to evaluate the evaporation dynamics *V*(*t*) of the water phase (Fig. 2g). We observe that the total evaporation time $t_f$ of the aqueous phase of emulsion drops depends on the wetting properties. The higher the surface wettability, the lower the total evaporation time. This is because for evaporating drops the evaporation rate is proportional to the drop radius, and since the radius of a drop is larger on a more wetting surface, the evaporation is faster^30^. As the contact angles remain constant for the most part of the evaporation process on all surfaces, we consider a simple diffusion-based evaporation model. By assuming that the rate-limiting step for evaporation is diffusion of water through the vapor phase, the volume *V* loss per unit time is proportional to the drop radius, i.e. $V \propto R^3$ for a constant contact angle and hence *V* should scale at $t^{3/2}$. This predicts that plotting $(V/{V_{0}})^\frac{2}{3}$ as a function of $t/t_f$ to yield a straight line^4,5^. Figure 2g (inset) shows that the evaporation dynamics of the water phase of this complex emulsion system for the most part indeed obey a simple vapor diffusion model.

### Confinement and coalescence of oil droplets

To further elucidate the underlying mechanisms causing drying emulsion drops on wetting surfaces to form ring-like deposits, we employ bright field microscopy to track events at the contact line right after drop deposition (Fig. 3). Zooming in on the drop boundary during the initial stage of evaporation reveals that, on the more hydrophilic substrates, oil droplets are pushed away from the contact line towards the drop center (Fig. 3b,c). This inward motion is the origin for the ring-like shapes observed in the microscopy experiments discussed above (Fig. 1b–d), and is also a prerequisite for the height increase at the drop center observed in the video microscopy experiment (Fig. 2b,c top panel,e). On wetting surfaces, the ratio of drop height to initial height remains either constant or in the order of unity (Fig. 2e). This is because, after contraction, the ring-like shape contains the phase-separated oil phase and inhibits its flow to the initial contact line. The width of these depletion zones increases roughly linearly in time for the two hydrophilic surfaces, but does not form on the hydrophobic one (Fig. 3d). We define the depletion zone to be fully formed as the oil droplet movement ceases. We suggest that the formation of depletion zones originates from strong capillary forces caused by geometrical constraint of oil droplets at the contact line, as previously observed in evaporating colloidal suspensions^15,31^ and drying-induced salt crystallization^14^. For the latter, growing NaCl crystals in evaporating salt water drops are pushed towards the center when they no longer fit in the corner of the drop near the contact line. Here, a similar scenario is observed where the oil droplets are excluded from a region on the order of the size of the oil droplets, which in the corner become confined between the free surface and the solid substrate (Fig. 3, illustrations). When the contact line recedes it sweeps the oil drops with it, leaving an aqueous film that subsequently evaporates and forms a layer of crystalline SDS. Geometrical considerations suggest that the length of the depletion zone is a function of both droplet size and contact angle^14,15,31^. As we deposit the same emulsion system on all surfaces, the droplet size remains constant. For this experiment we measured the contact angles separately, due to the small lag time between drop deposition and measurement in the video microscopy measurement. We observed that the contact angles only change marginally in the initial regime of evaporation (Fig.3d inset). Thus we find the depletion zone length to vary linearly with time as $L/L_0 \propto (a/\theta )\, t$ with *a* being dependent on drop size, but not surface wettability (Fig. 3). Using only 1 global fitting parameter allows us to estimate the evolution of the depletion zone on both wetting surfaces. In addition, given the high polydispersity of the oil droplets ($\sim$ 30%) we observe that smaller oil droplets segregate from larger once close to the contact line (Fig.3b,c), similar to what has been shown before in drying drops containing latex particles^31^.

At the complete formation of the depletion zones on the two more hydrophilic substrates, and around the onset of CCA evaporation on the hydrophobic substrate, we observe emulsion destabilization initiating at the contact line. As the water evaporates the oil droplets concentrate and jam followed by coalescence (see Fig. S2 in the Supplementary Information). The mechanisms of emulsion coalescence are still under debate. Explanations to describe this phenomenon include capillary pressures exceeding disjoining pressures^29,32^, involve topological rearrangements of emulsions oil droplets separated by thin films^33,34^, film breakage being purely stochastic processes^35^ or stretching-induced breakage followed by coalescence cascades^36^. In the case presented here, the emulsion drops destabilize in a similar manner as evaporating emulsion films^28,29,36^, in which the destabilization happens below a critical volume fraction of the continuous phase. The emulsion films simply become too thin, and break, leading to avalanche-type coalescence dynamics, as recently observed for via squeeze flow destabilized oil-in-water emulsions^36^.

To sum up, the formation of the ring-like structures presented in Fig. 1b–d can now be understood as follows: after emulsion drop deposition on untreated partial-wetting substrates, capillary forces oppose radial outward flows from the drop center and form areas depleted of oil droplets. As the water evaporates, SDS crystals precipitate within the depletion zones forming a coffee-ring like deposit. On the hydrophilic substrate (lowest initial contact angle), the depletion zone is wider and its formation faster than on the partial-wetting substrate. Conversely, on hydrophobic surfaces, any reversed motion is absent, as the comparatively high contact angle and lack of confinement eliminate capillary forces acting on the oil droplets. Thus, no ring-like depletion zone formation at the periphery can be observed, and phase-separated oil can freely spread on the substrate (Fig. 1a).

## Discussion

Drying emulsion drops exhibit a uniquely rich phenomenology, owing to their tendency to destabilize. We find that the deposition patterns strongly correlate with the initial contact angle caused by different degrees of wettability of the substrates. We observe two distinct scenarios: first for low contact angles, capillary forces induced by geometrical confinement enable the formation of depletion zones. Secondly, for high initial contact angles, the formation of depletion zones is inhibited and causes an oil film to spread evenly on the surface upon destabilization, thus suppressing the coffee-ring like deposit found on wetting glass-slides. This effect and its reversal can be beneficial in a variety of applications, for example in cosmetics or medical delivery systems, where sometimes a controlled release of oil from emulsions is desired^37^. Emulsion-type creams are recognized as particulate carriers for topical drugs given their ability to allow passage through the skin barrier^38^. For this reason, controlling the deposits on human skin, which is generally hydrophobic^39^, can prove vital for medical applications. Considering the evaporation dynamics, a simple diffusion model explains the time-dependent evolution of the drop volume as a function of time.

## Materials and Methods

### Emulsion preparation

Our experiments were performed on an emulsion consisting of oil droplets dispersed in water and stabilized by surfactant. The emulsion was prepared by mixing 500 cst silicon oil (from Sigma-Aldrich) with a continuous phase containing 1% (mass fraction) of the ionic surfactant SDS (from Sigma-Aldrich) in pure water (MilliQ) using the viscous-phase method. As a mixer, we used a Silverson L5M-A. Oil was slowly added to the continuous phase while mixing at 6000 rpm for 20 min to obtain a initial o/w volume fraction of 0.80. This technique yields an average oil droplet size of $\sim 10 \, \upmu \hbox {m}$, with a polydispersity of $\sim$ 30%. The emulsion was then further diluted with aqueous phase containing Rhodamine 6G (R6G) at $1 \, \upmu \hbox {M}$ and SDS of 1% mass fraction to produce the final samples of 0.60 volume fraction for the optical, confocal and video microscopy measurements. When stored without evaporation, this emulsion was stable for several months.

### Substrate preparation

In order to introduce hydrophilic functional groups on the surface of glass slides, a Zepto Electronic Diener plasma cleaner was used^40^. Glass slides were treated for $\sim$ 60 s in a low-pressure plasma chamber. To study emulsion drop evaporation on hydrophobic surfaces, a special surface silanization technique was used. Prior to the silanization process plasma-treatment was applied to clean the surfaces, followed by 10 min of immersion into a mixture of 1 mL trichloro (octyl)-silane in 99 mL toluene. The glass slides were cleaned with ethanol and deionized water prior use. Table 1. shows initial contact angles for all emulsion drops and neat silicon oil and water drops as references.

**Table 1:** Contact angles for emulsion, neat water and silicon oil drops on different substrates.

	Emulsion	Water	Silicon oil
Hydrophobic	55.9	96.5	18.8
Partial-wetting	22.9	43.6	13.2
Hydrophilic	9.7	9.7	7.6

### Sobel edge-detection

For the edge detection analysis, as depicted in Fig. 1d, we employed a Sobel-edge detection algorithm that calculates sharp changes within the intensity of the images by computing horizontal and vertical derivatives, thus highlighting structural heterogeneities. The algorithm was used within the software package imageJ^41^.

### Evaporation dynamics experiment

Evaporation experiments presented in Fig. 2 were carried out on a home-built setup comprising confocal microscopy and video microscopy at a relative humidity of $\sim$ 40%. We used confocal microscopy to visualize the emulsion structure from below, and side-view video microscopy to follow the drop profile and determine droplet base diameter, height, contact angle and volume. R6G was excited at 514 nm and emitted light was collected between 530 and 600 nm. Experimental conditions limited the time between drop deposition and measurement to $\sim$ 100 s.

### Contact line dynamics experiment

Contact line dynamics experiments to demonstrate confinement and coalescence of oil droplets were carried out using a bright field microscope with 40x magnification. Here, the time between drop deposition and measurements were $\sim$ 15 s. Contact angles corresponding to this initial regime were recorded using a Drop Shape Analyzer from Krüss.

### Raman microscopy

The aforementioned sample preparation procedure was repeated for the Raman microscopy measurements, although without the addition of R6G in order to prevent interfering fluorescence. The Raman signal was measured on a confocal Raman microscope (Witec, Alpha 300 R) coupled to a CMOS camera (Andor, Newton EMCCD, DU970P-BVF-355). The laser wavelength used was 532 nm with a grating of $600 \, {\hbox {g mm}}^{-1}$. Each spectrum was averaged 25 times with an integration time of 30 s.

## Supplementary information

Supplementary information 1.

Supplementary information 2.
