# Author Correction: Deposits from evaporating emulsion drops

**DOI:** 10.1038/s41598-020-74408-y

**Published:** 2020-10-08

**Authors:** M. R. Bittermann, A. Deblais, S. Lépinay, D. Bonn, N. Shahidzadeh

**Affiliations:** Van der Waals-Zeeman Institute, IoP, Science Park 904, Amsterdam, Netherlands

Correction to: *Scientific Reports* https://doi.org/10.1038/s41598-020-71964-1, published online 10 September 2020

In this Article, the legend of Figure 2 is incorrect:

“Scale bar is 160 μm”.

should read:

“Scale bars are 1 mm”.

Additionally, in the Results section under the subheading, ‘Confinement and coalescence of oil droplets’,

“At the complete formation of the depletion zones on the two more hydrophilic substrates, and around the onset of CCA evaporation on the hydrophobic substrate, we observe emulsion destabilization initiating at the contact line”.

should read:

“At the complete formation of the depletion zones on the two more hydrophilic substrates, and around the onset of mixed mode evaporation on the hydrophobic substrate, we observe emulsion destabilization initiating at the contact line”.

