# Supplementary material for: Deposits from evaporating emulsion drops
Source: Sci Rep. 2020 Sep 10;10:14863. doi: 10.1038/s41598-020-71964-1 (PMC7483418; doi:10.1038/s41598-020-71964-1)
Supplement: Supplementary file 1 — Supplementary information 1. [file 41598_2020_71964_MOESM1_ESM.pdf]

# Supplementary Information for Deposits from evaporating emulsion drops

M. R. Bittermann, A. Deblais, S. Lépinay, D. Bonn, N. Shahidzadeh

## Raman microscopy

To study the exact composition of the individual features of evaporated emulsion drops, we employed Raman microscopy. By comparing the spectra of neat silicon oil and crystalline SDS to the spectra obtained by focusing on both bulk and ring regions of dried emulsion drops on different surfaces, we observed that the ring-like shapes consist of crystalline SDS and silicon oil (Fig. S1). On the other hand, we found that the oil film released by drying on a hydrophobic substrate is devoid of surfactant, which accumulates in the drop center instead.

## Coalescence

The lack of scattering in the dried emulsion drops as shown in the profilometer experiments (Fig. 1, a-c bottom panel) suggests oil droplet destabilization by coalescence. We confirmed this assumption by carrying out additional confocal fluorescence microscopy using a high magnification objective. Fig. S2 shows that coalescence events not only take place at the drying front, but also in the bulk. Similar behavior has been observed in drying emulsion films.[1]

## Evaporation dynamics - volume calculation

Small drops, in which surface tension dominates gravity assume the shape of a spherical cap. The volume V of a spherical cap can be calculated from its base radius and contact angle

$$V = \beta(\frac{r_b}{sin(\theta)})^3 \frac{\pi}{3} \tag{1}$$

with $\beta$ being dependant on $\theta$ only

$$\beta = (1 - cos(\theta))^2 (2 + cos(\theta)) \tag{2}$$

A drop evaporating diffusively in CAA mode can be described by[2,3]

$$V = V_0(1 - \frac{t}{t_f})^{\frac{3}{2}} \tag{3}$$

with $V_0$ being the initial drop volume and $t_f$ the final evaporation time.

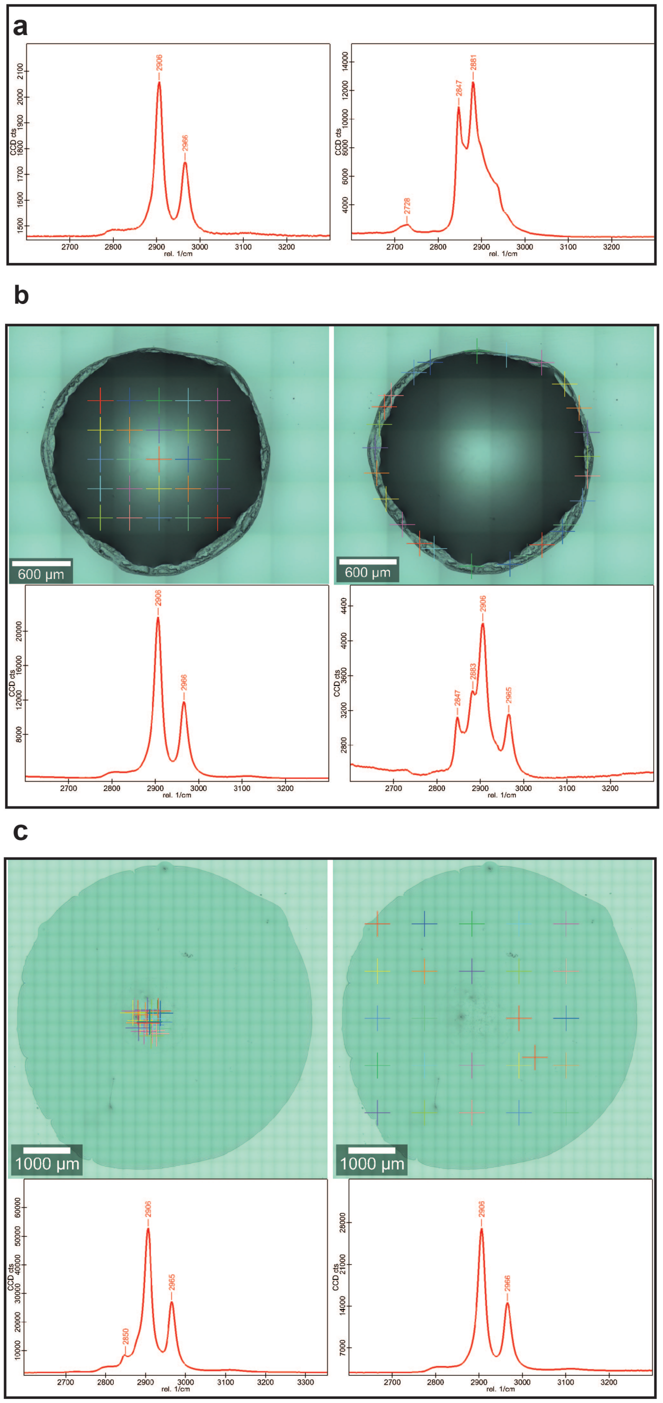


Figure S1: Raman microscopy measurements of evaporating emulsion drops on a wetting and hydrophobic surface. (**a**) The reference spectra for silicon oil (left) and crystalline SDS (right) can be used to identify deposits of the dried drops via differences in the $CH_2$-stretching modes in the region 2700-3000 $cm^{-1}$. Silicon oil is characterized by peaks at 2906 $cm^{-1}$ and 2966 $cm^{-1}$ and SDS at 2847 $cm^{-1}$ and 2881cm $^{-1}$. The sample spectra were recorded at the center (left) and periphery (right) of: (**b**) Dried drops on partial-wetting surfaces. The spectra show crystalline SDS to appear within the depletion zone. (**c**) Dried drops on hydrophobic surfaces, where SDS can be detected only within the drop center.

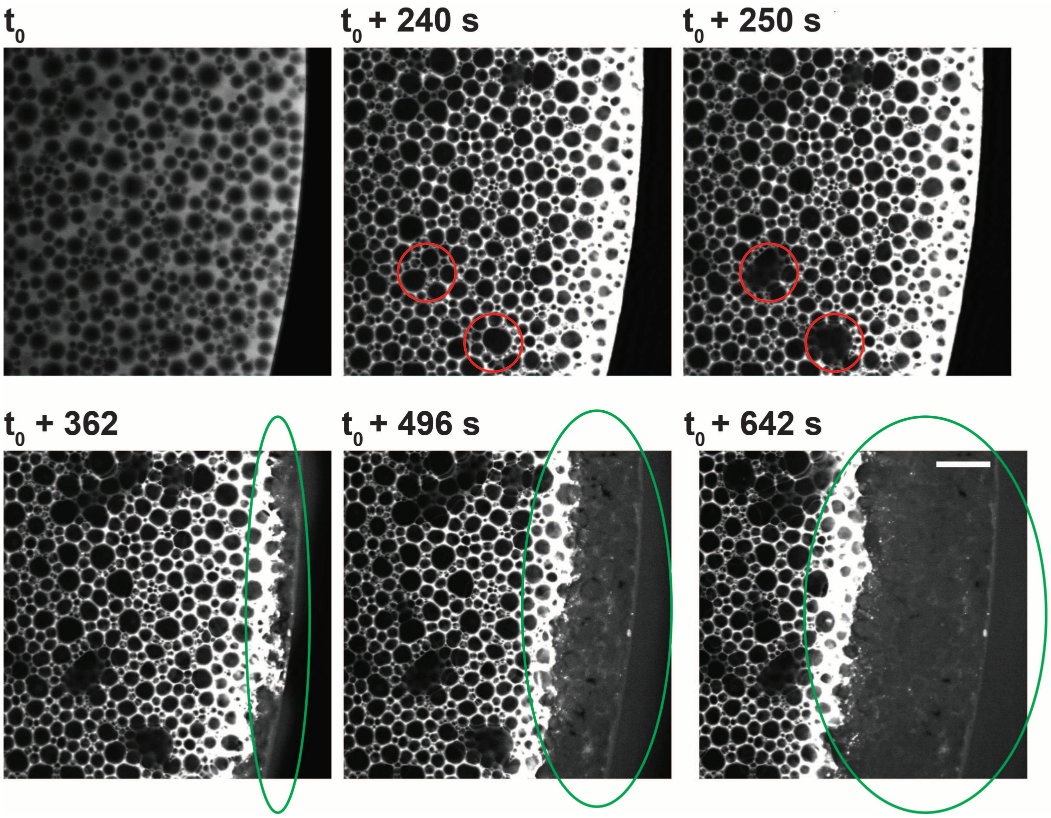


Figure S2: Confocal fluorescence microscopy images taken during the initial stages of a drying emulsion drop on a hydrophobic surface. All images were recorded close to the contact line. Following drop deposition the oil droplets jam as the water evaporates. Here, we observed both bulk (red highlights) and front (green highlights) coalescence. Scale bar is 30 $\mu$m.

# References

[1] Feng, H. *et al.* Two modes of phase inversion in a drying emulsion. *Soft Matter* **9**, 2810–2815 (2013).

[2] Picknett, R. & Bexon, R. The evaporation of sessile or pendant drops in still air. *Journal of Colloid and Interface Science* **61**, 336 – 350 (1977).

[3] Erbil, H. Y., McHale, G. & Newton, M. I. Drop evaporation on solid surfaces: Constant contact angle mode. *Langmuir* **18**, 2636–2641 (2002).
